# Life review on psychospiritual outcomes among older adults with life-threatening illnesses: A systematic review and meta-analysis

**DOI:** 10.3389/fpsyt.2023.1077665

**Published:** 2023-02-28

**Authors:** Mandong Liu, Ying Wang, Yan Du, Iris Chi

**Affiliations:** ^1^Institute of Social Development, Southwestern University of Finance and Economics, Chengdu, China; ^2^School of Philosophy and Sociology, Lanzhou University, Lanzhou, China; ^3^Evidence-Based Social Science Research Center of Lanzhou University, Lanzhou, China; ^4^School of Nursing, The University of Texas Health Science Center at San Antonio, San Antonio, TX, United States; ^5^Suzanne Dworak-Peck School of Social Work, University of Southern California, Los Angeles, CA, United States

**Keywords:** life review, psychospiritual well-being, life-threatening illness, quality-of-life, older adult, anxiety, depression

## Abstract

**Background:**

At the intersection of old age and illness, older adults with life-threatening illnesses (LTI) are a group who often show resilience and seek validation of life, acceptance, and integration of past and now, even under the fear of loss, suffering, and dying evoked by life adversities. Life review has been widely conducted to help older adults enhance well-being and cope with burdens. Spirituality is an important part of an older adult’ overall well-being, especially for those with LTI. However, few review studies examined the effectiveness of life review interventions on psychospiritual outcomes among this population. The aim of the study was to examine the effectiveness of life review on psychospiritual well-being among older adults with LTI.

**Methods:**

A systematic review with meta-analysis following the recommendations of the Cochrane Collaboration was conducted. Database searches included PubMed, PsycINFO, the Cochrane Library, the Campbell Library, EBSCO, CNKI, and the Airiti Library up to March 2020. Gray literature and reference lists from relevant articles were also searched and reviewed.

**Results:**

In total, 34 studies were included in the systematic review and the meta-analysis for outcomes of depression (*n* = 24), quality-of-life (QOL) (*n* = 10), anxiety (*n* = 5), life satisfaction (*n* = 3), mood (*n* = 3), apathy (*n* = 2), and general well-being (*n* = 2). Other psychospiritual outcome measures included spirituality, self-esteem, meaning in life, hope, and some multi-dimensional instruments. The studies greatly varied in program design, content, format, length, and more. Although with high heterogeneity, meta-analysis results demonstrated standardized mean differences in favor of life review in decreasing depression, anxiety, negative mood, and increasing positive mood and QOL compared with the control group.

**Conclusion:**

This review calls for including more psycho-spiritual well-being measures among interventions for older adults with LTI, as well as studies with rigorous designs in future research.

## Introduction

As age increases, people are more likely to have one or more health-related problems, including life-threatening illnesses (LTI), such as cancer and heart diseases (1). Living with LTI causes pain and distress and transitions in one’s physical, psychological, social, and spiritual aspects of life (2, 3). Many people living with LTI show remarkable resilience and gradually yearn for a life larger than illness and one’s physical conditions (4), indicating the importance of exploring this population’s psychospiritual well-being.

Gleig (5) defined psychospiritual well-being as the integration of psychological and spiritual well-being. It includes embracing a spiritual dimension of the human being as fundamental to psychic health and full human development, and using methods in both fields in a holistic approach for inner growth. Researchers further pointed out that such outcomes could include self-awareness, coping and adjusting effectively to stress and loss, having satisfying relationships and connectedness with others, a sense of faith, a sense of empowerment and confidence, and living with meaning and hope, etc. (2, 4, 6, 7); Lin and Bauer-Wu (8). The key to understand one’s psychospiritual well-being is to hold a holistic, integrated view of healing and inner growth.

To enhance the overall well-being of people facing challenges associated with LTI, pharmacological and non-pharmacological interventions have been increasingly developed and delivered. One of the non-pharmacological interventions is life review (9, 10). Life review has been widely used among different populations to achieve therapeutic benefits such as enhancing one’s quality-of-life (QOL) and decreasing anxiety and depression (11). Older adults are very likely to experience various types of adversities, such as deteriorating health, worsening financial security, and illness or death of a family member. In the phase of coping (or not coping), some older individuals fail to adapt to the aging process, while others embrace opportunities and actively engage themselves in challenges, viewing their lives and health as satisfactory (12). According to Luthar et al. (13), it may be easier to detect resilient attitudes, activities, and behaviors in later life, as compared to younger individuals, older adults have acquired more life experiences to perform certain coping strategies to achieve positive outcomes; among these strategies, life review is a promising one (14).

Although sometimes used interchangeably with reminiscence by researchers and practitioners, life review and reminiscence bear significant differences: reminiscence involves recalling a memory, and thus the main goal is to stimulate one’s cognitive functioning, while life review involves reflecting and evaluating what the memory means to the person, and the main goal is usually to improve QOL and achieve ego-integrity (15–17). Some systematic review and meta-analysis studies separated the two [e.g., (18)], while others included both [e.g., (19)]. Life review definitions used by researchers were inconsistent. For example, in Ning et al. (20), although called group reminiscence, stroke patients participated in structured conversations on “First Met,” “Unforgettable Youth,” “My Other Half,” “My Favorite Entertainments,” “My Pride,” and “My Late-Life” with a main purpose to alleviate depressive symptoms, instead of focusing on cognition training. In the current study, we included studies that delivered an intervention of recalling and/or reflecting on memories, no matter how the authors named the intervention. Together we call this type of intervention “life review.”

Several systematic review and meta-analysis studies have been conducted to evaluate the effects of life review. Most of these studies focused on general older adult populations [e.g., (21, 22)] and people with cognitive impairments [e.g., (23, 24)]; the outcome measures focused on QOL, depression, and cognition [e.g., (11, 19)]. Not enough attention was paid to how life review could benefit older adults with LTI on other psychospiritual outcomes (e.g., hope, self-esteem). At the intersection of old age and illness, older adults with LTI are a group who often seek validation of life and acceptance and integration of past and now, even under the fear of loss, suffering, and dying evoked by LTI (4, 25). This process of seeking inner strength is highly consistent with the goals and procedures of life review. Thus, examining how life review could psychospiritually benefit older adults with LTI is essential and urgent.

In a previous study, Chen et al. (26) conducted a systematic review of the effects of life review on psychospiritual well-being among patients with LTI. However, they only included patients with cardiovascular disease, cancer, acquired immune deficiency syndrome (AIDS), and organ failure. Additionally, with 11 studies included, they only included depression, QOL, and self-esteem in the meta-analysis; nor did they clearly define what psychospiritual well-being is. The current systematic review and meta-analysis study aimed to thoroughly examine the effects of life review interventions on psychospiritual outcomes among older adults with LTI.

## Methods

This systematic review and meta-analysis study was conducted following the guidelines outlined by the Preferred Reporting Items for Systematic reviews and Meta-Analyses (PRISMA) Group (27).

### Criteria for considering studies for this review

#### Participants

Older adults with at least one diagnosed LTI or condition were included. In this study, we defined LTI based on the top 10 causes of death recorded by the World Health Organization (28) for upper-middle- and high-income countries (as based on literature review for regions of potentially included studies). After combining different types of cancer, these include cancer, ischemic heart disease, stroke, chronic obstructive pulmonary disease, lower respiratory infection, diabetes mellitus, kidney disease, and Alzheimer’s disease and related dementias (ADRD). Additionally, we included studies on palliative or hospice older adult patients, as having a life-threatening condition is one criterium to receive such service. If a study contains participants with more than one disease or in more than one age group, only those that separate participants’ conditions and age groups for analysis were included.

#### Interventions

Interventions needed to meet our definition of life review: an intervention of recalling and/or reflecting on memories, no matter how the authors named the intervention. There was no requirement for minimum or maximum intervention duration, location, or interventionist. However, multi-component interventions where life review was only part of the intervention were excluded.

#### Control

There was no requirement for control: a control could be no treatment, treatment as usual, waitlist, passive control (social contacts and conversations unrelated to life review, such as on social security income, diet), and active control (counseling about one’s life but not structured life review sessions; also could be another psychosocial intervention). However, for the meta-analysis, considering the huge effects an active control can have on the outcome, it was not included in the meta-analysis. In other words, only groups of no treatment, treatment as usual, waitlist, and passive control were used for meta-analysis.

#### Outcome measures

Studies that assessed any psychospiritual effect of a life review intervention were included in the systematic review, provided that standardized assessments, rating scales, or questionnaires were used. These outcomes included but not limiting to anxiety, depression, QOL, life satisfaction, mood, hope, dignity, meaning in life, spiritual well-being, etc. Outcomes measured at post-intervention and additional follow-up were considered. Outcomes that were measured in at least two studies were included in the meta-analysis.

#### Types of study

Studies that used random assignment with pre- and post-intervention assessments were considered for this review. These included RCTs, defined as studies in which individual participants are randomly assigned to either an intervention group or to a control group (29). Although some studies did not explicitly used the term “RCT,” we still included them if they could not be ruled out using a randomized controlled design (e.g., authors did not explicitly use the term “RCT” but mentioned “randomized” and “controlled.” An example description is: “Participants were randomized into two groups”).

#### Language

Study was written in English or Chinese with full-text available.

### Search methods for identification of studies

#### Database searches

The following academic databases were searched in March 2020: PubMed; PsycINFO; the Cochrane Library; the Campbell Library; EBSCO; CNKI; and the Airiti Library. Search terms were: (“old*” OR “elder*” OR “age*” OR “aging” OR “geriatric*” OR “senior” OR “late life”) AND (“life review” OR “reminiscence” OR “dignity therapy” OR “meaning-centered therapy” OR “life story” OR “storybook” OR “memoir” OR “life history” OR “life experience” OR “autobiograph*” OR “life album” OR “memory book” OR “memory album” OR “narrative therapy”) AND “random*” OR “rct”). Search terms were customized to each database.

#### Gray literature

The following websites were searched for gray literature: Google Scholar, ProQuest Dissertations & Theses Database (PQDT), and Duxiu. Authors of relevant conference abstracts were reached out for possible information sharing.

#### References

We searched the reference lists of included articles (i.e., articles eligible for further analysis) for additional relevant studies.

### Data collection and analysis

#### Screening

Reviewers ML and YW each screened half of the titles and abstracts. For abstracts with no full-text, authors’ correspondence information was searched online, and emails were sent to request the full-text of the study, if one existed. After that, full-text was retrieved for articles passing the abstract screening stage for further screening. Four reviewers were divided into two groups, with each group screening half of full-text articles using a pre-designed Google form with inclusion and exclusion criteria such as illness and outcome measures. Any uncertainties concerning suitability were discussed at weekly group meetings with all four investigators: Before a weekly group meeting, the first author compared the results and informed the whole group of each pair’s inconsistencies, and then asked the other pair to screen those articles again, so that all four of us could discuss inconsistencies in the group meeting. Full-text of all articles can be provided per request.

#### Data extraction

Four reviewers, again in two groups, independently extracted data using a pre-designed Google form, which was pilot tested with four articles. Each group extracted data from half of included studies. The following data were extracted, and where necessary, additional information was requested from authors:

(1) Basic study information: authors, reference, country/region.
(2) Participant characteristics: illness/condition, total number and number in each group, age, gender, race/ethnicity.
(3) Intervention characteristics: intervention content, individual or group format, in-person or virtual, setting, length (e.g., number of weeks), number of sessions, duration per session, control/comparison.
(4) Intervention assessment information: time point (e.g., pretest, posttest, follow-up), measures, outcomes with screenshots (including the mean and standard deviation of final values, and number of participants in each group at each time point), outcome raters (e.g., patients, caregivers, staff).

After comparing results within a group, any uncertainties that could not be solved were discussed in weekly meetings with all four reviewers: Before a weekly group meeting, the first author compared the results and informed the whole group of each pair’s inconsistencies, and then asked the other pair to read the relevant part of the articles, so that all four of us could discuss inconsistencies in the meeting.

### Data analysis

RevMan 5.4 was used for meta-analysis. Outcomes measured in at least two studies were included in the meta-analysis. Heterogeneity was assessed using an I^2^ statistic. To interpret heterogeneity, reviewers followed Cochrane guidance (i.e., 0–40% might not be important; 30–60% may represent moderate heterogeneity, 50–90% may represent substantial heterogeneity; and 75–100% is considerable heterogeneity) (30). Where there were high levels of heterogeneity of the treatment effect between studies, a random-effects model and the standardized mean difference were used. If one study used more than one instrument to measure the same outcome variable, the team used the more commonly used instrument for the analysis. Subgroup and sensitivity analysis were performed if the heterogeneity was high. Where necessary, we performed subgroup analyses with various possible reasons for heterogeneity, including study quality (one analysis included studies with low random allocation risk, and another analysis excluded studies with four or more “high” plus “unclear” ratings in ROB appraisal), cognition (only included ADRD patients), measurement tool (compared meta-analysis results of studies that used different measurement tools for the same outcome), outcome rater (compared results of studies where outcomes were rated by older adults themselves or by caregivers), control group (compared results of studies that used different control groups, such as usual care vs. no intervention), country/region (compared results of studies that were conducted in different countries/regions), intervention weeks (>10 weeks vs. ≤10 weeks), number of intervention sessions (>10 sessions vs. ≤10 sessions).

### Assessment of risk of bias in included studies

Four reviewers independently assessed the risk of bias using the Cochrane “Risk of Bias” tool (31), classifying each category as low, high, or unclear ROB. Any disagreements regarding ROB ratings were discussed at weekly meetings until a consensus was reached.

## Results

### Search results

**FIGURE 1:**
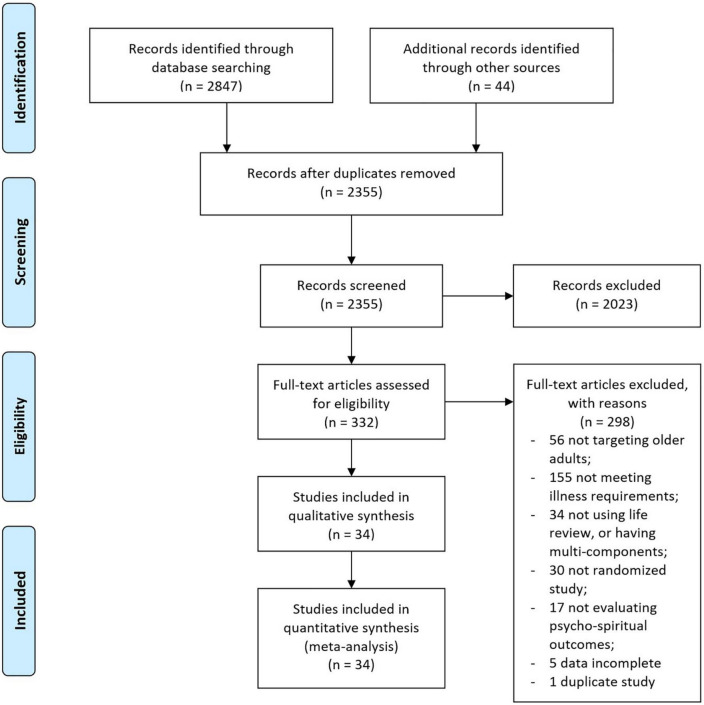
Flowchart of the study selection process.

Figure 1 shows the PRISMA flowchart of the study review and selection process. A total of 34 studies, representing a total of 2,752 participants, were included in the review and the meta-analysis.

### Study characteristics

Table 1 presents the characteristics of included studies. Among 34 studies, 19 were conducted in Asia (13 in mainland China, three in Taiwan, two in Japan, one in Hong Kong), 11 in Europe (five in the United Kingdom, three in Turkey, two in Belgium, one in Portugal), three in the United States, and one in Argentina. Eighteen were RCT, 14 were randomized studies, and two were “quasi-experimental” studies, as claimed by authors, in which the randomization could not be ruled out.

**TABLE 1:** Characteristics of included studies.

Study references and country/Region	Participants and # of allocation	Study design	Intervention group (IG)	Control group (CG)	Outcome measures	Data collection time points and raters	Analysis	Between-group results
(37) USA	≥55 y/o palliative care patients and caregivers from community and assisted living *n* = 45 dyads	RCT	Patient-caregiver reminiscence, length unclear *n* = 22 dyads	Supportive phone calls *n* = 23 dyads	Depression: CES-D Positive and negative affect: PANAS (positive, negative) Physical and psychological symptoms and distress: MSAS-SF (physical symptoms, physical bother, emotional symptoms, emotional bother) Spirituality: BMMRS (daily spiritual experiences, forgiveness, religious meaning, abandonment) Meaning in life: Meaning in Life Scale	Pre, post, f/u (13–14 weeks after post) Rated by patients	Unclear	IG reported greater reduction in emotional symptoms (*P* = 0.02), emotional bother (*P* = 0.04), abandonment (*P* = 0.037), and greater improvement in forgiveness (*P* = 0.031). Others: NS
(60) USA	≥60 y/o ADRD patients from nursing homes *n* = 51	RCT	6-week 12-session group reminiscence, environmental support, and individualized behavioral activities based on reminiscence *n* = 26	Usual nursing home activities such as singing, bingo *n* = 25	Depression: CSDD, GDS Quality-of-life: QOL-AD	Pre, post CSDD and GDS rated by patients; QOL-AD rated by patients and nursing home staff for a weighted score	2 × 2 ANOVA	CSDD: IG showed greater reduction, F(1, 49) = 13.43, Cohen’s *d* = 1.01 GDS, QOL-AD: NS
(49) Turkey	≥60 y/o ADRD patients from elderly care and rehabilitation centers *n* = 66	Quasi-experimental*	12-week 12-session group reminiscence *n* = 33	Weekly conversations on religious or official special days in the relevant week, health, or current issues *n* = 33	Depression: GDS	Pre, post Rated by patients	Repeated measures ANOVA	GDS: IG showed greater reduction, *F* = 30.518, *P* < 0.001
(45) UK	60–99 y/o ADRD patients from assisted living facilities *n* = 31	Randomized study**	6-week 6-session individual life review *n* = 15	Usual care *n* = 16	Depression: CSDD Mood: AMS (positive, negative)	Pre, post Rater unclear	MANCOVA	CSDD: S, *F* = 7.54, *P* = 0.015 AMS positive: S, *F* = 9.47, *P* = 0.008 AMS negative: NS
(58) Taiwan	60–95 y/o ADRD patients from nursing homes *n* = 66	Randomized study	12-week 12-session group reminiscence *n* = 33	Usual care *n* = 33	Depression: GDS Apathy: AES-C (behavior, cognition, emotion) Multi-dimension: NPI-Q (reported apathy and depression)	Pre, post GDS rated by patients, AES-C and NPI-Q rated by staff	Wilcoxon signed-rank test	GDS: S -, *Z* = −2.99, *P* = 0.003 AES-C behavior: S -, *Z* = −3.10, *P* = 0.002 AES-C cognition: S -, *Z* = −1.95, *P* = 0.050 NPI-Q depression: S -, *Z* = −2.20, *P* = 0.028 AES-C emotion, NPI-Q apathy: NS
(41) Mainland China	63–80 y/o ADRD patients from a hospital *n* = 90	Randomized study	Individual reminiscence, length unclear *n* = 45	Usual care: routine care, cognitive training *n* = 45	Quality-of-life: QOL-AD Depression: CSDD	Pre, post Rater unclear	*t*-test	CSDD: S -, *P* < 0.05 QOL-AD: S +, *P* < 0.05
(32) Mainland China	60–75 y/o stroke patients from a hospital *n* = 83	Randomized study	12-week 12-session group reminiscence *n* = 42	Usual care: health education, routine care, rehab *n* = 41	Depression: HAMD multi-dimension: NOSIE (only reported overall score)	Pre, post Rated by two raters	*t*-test	NOSIE: S +, *t* = 3.509, *P* < 0.01 HAMD reported post M (SD) but did not report between group results
(55) HK	ADRD patients from old age homes *n* = 101	RCT	6-week 6-session individual reminiscence *n* = 36	Participants discussed “diet and health,” “social security for the elderly,” etc. *n* = 35 No treatment *n* = 30	Well-being: WIB	Pre, post, f/u (6 weeks after intervention) Rated by raters	GLM with repeated measures	NS
(61) Mainland China	>60 y/o ADRD patients from a hospital *n* = 84	Randomized study	12-week 12-session individual reminiscence *n* = 42	Usual care: medication, routine care, diet, exercise, cognitive training *n* = 42	Depression: CSDD Quality-of-life: QOL-AD	Pre, post Rated by psychologists	*t*-test	QOL-AD: S +, *t* = 8.175, *P* < 0.01 CSDD: S -, *t* = 5.320, *P* < 0.01
(35) Mainland China	≥60 y/o ischemic heart disease patients from a hospital *n* = 80	Randomized study	Individual narrative therapy, length unclear *n* = 40	Usual care: health education on constipation, diet *n* = 40	Anxiety: SAS	Pre, post Rater unclear	*t*-test	SAS: S -, *t* = 2.495, *P* = 0.015
(33) Mainland China	Older stroke patients from a hospital *n* = 90	Randomized study	4-week 4-session individual reminiscence *n* = 45	Usual care: communication about source of depression, health education, medication *n* = 45	Depression: HAMD	Pre, post Rater unclear	*t*-test	HAMD: S -, *t* = 26.747, *P* < 0.001
(56) Mainland China	≥65 y/o ADRD patients from a hospital *n* = 90	RCT	12-week 24-session group reminiscence *n* = 45	Usual care: medication, routine care *n* = 45	Depression: CSDD Multi-dimension: NPI-Q (only reported overall score)	Pre, during intervention (4 weeks after intervention began), post (12 weeks), f/u (24 weeks) Rated by formal or family caregivers	*t*-test	CSDD: S -, post: *t* = 2.076, *P* < 0.05; f/u: *t* = 3.834, *P* < 0.05 NPI-Q: NS
(47) Mainland China	60–88 y/o COPD patients from a hospital *n* = 80	Randomized study	4-week 8- to 12-session individual narrative therapy + usual care *n* = 40	Usual care: medication, psychological care, diet, education *n* = 40	Anxiety: SAS Depression: SDS	Pre, post Rater unclear	*t*-test	SAS: S -, *t* = −11.589, *P* = 0.002 SDS: S -, *t* = −9.025, *P* = 0.003
(51) Turkey	ADRD patients from a nursing home *n* = 60	RCT	8-week 8-session group reminiscence *n* = 30	No intervention *n* = 30	Quality-of-life: QOL-AD Depression: CSDD	Pre, post CSDD rated by a researcher based on info from patients and caregivers; QOL-AD rated by patients and caregivers for a combined score	One-way ANOVA	QOL-AD: S +, *F* = 0.748, *P* < 0.001 CSDD: S -, *F* = 0.637, *P* < 0.001
(62) Portugal	ADRD patients from nursing homes *n* = 41	Quasi-experimental	5-week 5-session individual reminiscence *n* = 20	Usual care *n* = 21	Anxiety: GAI Depression: CSDD, GDS-5	Pre, post CSDD rated by nursing staff; GDS-5 and GAI rated by patients	Mann–Whitney test	GAI: S -, *Z* = 2.836, *P* < 0.01 GDS-5: S -, *Z* = 3.730, *P* < 0.001
(50) Turkey	≥65 y/o ADRD patients from nursing homes *n* = 32	RCT	12-week 12-session group reminiscence *n* = 16	Unstructured casual conversations on current events, health problems of older people, social security incomes, diet, and family visits *n* = 16	Apathy: AES	Pre, post Rated by patients	Mann–Whitney test	AES: S +, *Z* = −4.550, *P* = 0.001
(34) Mainland China	Older stroke couples *n* = 75 dyads	RCT	8-week 8-session patient-spouse reminiscence (G1) *n* = 25 dyads	8-week 8-session individual reminiscence attended by caregivers (G2) *n* = 22 dyads Usual care: routine health education (G3) *n* = 28 dyads	Life satisfaction: SWLS	Pre, post, 1 month after intervention, 3 months after intervention Rated by patients	3 × 4 ANOVA	SWLS: score was higher in G1 and G2 compared with G3, and G1 compared with G2 at T1, T2, and T3 (*P* < 0.001).
(42) UK	ADRD patients from care homes *n* = 17	Randomized study	At least 12-session individual life review *n* = 8	No intervention *n* = 9	Life satisfaction: LSI-A Depression: GDS-SF Self-esteem: RSE	Pre, post, f/u (6 weeks after intervention) Rated by patients	Repeated measures ANOVA, *t*-test	GDS-SF: significant differences between groups over time (*F* = 5.59, *P* = 0.009), but NS between groups at posttest and f/u. LSI-A and RSE: NS
(52) Japan	≥65 y/o ADRD patients from long term care facilities *n* = 36	RCT	6-week 6-session group making and eating rice balls when reminiscing in the past *n* = 17	Only eating rice balls *n* = 19	Depression: CSDD Multi-dimension: MOSES (self-care, disorientation, depression, irritability, withdrawal)	Pre, post Rated by occupational therapists	Mann–Whitney test	NS
(20) Mainland China	≥60 y/o stroke patients from community *n* = 86	RCT	6-week 6-session group reminiscence *n* = 43	Usual care: health education on diet, exercise, medication, psychological care *n* = 43	Depression: HAMD	Pre, post rated by patients	*t*-test	HAMD: S -, *t* = 4.71 *P* < 0.001
(63) Mainland China	68–88 y/o ADRD patients from a hospital *n* = 82	Randomized study	12-week 24-session individual reminiscence *n* = 42	Usual care, person-centered *n* = 40	Depression: GDS	Pre, post Rated by patients	*t*-test	GDS: S -, *t* = 6.118, *P* < 0.001
(43) Argentina	ADRD patients from nursing homes *n* = 135	RCT	12-week 24-session individual/group reminiscence *n* = 45	Active control: 12-week 24-session counseling and informal social contacts *n* = 45 Passive control: 12-week 24-session unstructured social contacts on topics of social security income, diet and family visits *n* = 45	Anxiety: RAID (results not reported) Depression: DRS (results not reported) Quality-of-life: self-reported QOL scale Well-being: WIB	Pre, post, 6-month f/u QOL scale rated by patients; WIB rated by raters	3 × 3 ANOVA	QOL Scale: group x time: mean square = 148.1, *F* = 14.7, *P* < 0.01 WIB: NS
(46) UK	ADRD patients from care homes *n* = 24	RCT	12-week 11–16-session individual life review *n* = 12	Gift group: relatives developed a life story book of patient without patient involvement *n* = 12	Depression: GDS-12R Quality-of-life: QOL-AD	Pre, post, 18-week f/u Rated by patients	One-way ANCOVA	QOL-AD: At post, IG showed significant improvement compared to CG, F(1, 20) = 5.11, *P* = 0.035 Others: NS
(57) Japan	ADRD patients from a geriatric health services facility, including Alzheimer disease (AD) and vascular dementia (VD) *n* = 60, AD = 24, VD = 36	RCT	8-week 8-session group reminiscence AD = 12, VD = 18	Usual care: mild exercises, meals, and bathing AD = 12, VD = 18	Multidimension: MOSES (self-care, disorientation, depression, irritability, withdrawal)	Pre, post, 6-month f/u Rated by family caregivers	Repeated measures ANCOVA	MOSES withdrawal: significant effects in VD IG (*P* < 0.001). VD IG had lower score than CG at post and f/u. Others: NS
(64) Mainland China	61–77 y/o ADRD patients from a hospital *n* = 74	Randomized study	12-week 12-session individual reminiscence *n* = 37	Usual care: medication and routine care *n* = 37	Quality-of-life: QOL-AD	Pre, post Rater unclear	*t*-test	QOL-AD: S +, *P* = 0.000
(38) UK	ADRD patients and caregivers from community *n* = 11 dyads	RCT	11 (for caregivers) + 7 (for dyads) weeks weekly reminiscence *n* = 7 dyads	No intervention *n* = 4 dyads	Quality-of-life: QOL-AD	Pre, post Rated by patients and caregivers	*t*-test for change post - pre	NS
(40) Belgium	65–100 y/o ADRD patients from long term facilities, day care centers, and psychiatric inpatient care facility *n* = 82	Randomized study	4-week 8-session individual reminiscence *n* = 41	Usual care *n* = 41	Depression: GDS-30, CSDD Multi-dimension: NPI-Q (only reported overall score)	Pre, post Rated by patients	Mann–Whitney test for change post - pre	GDS-30: S -, *P* < 0.001 CSDD: S -, *P* < 0.01
(53) Belgium	≥60 y/o ADRD patients from nursing homes *n* = 72	RCT	8-week 16-session individual reminiscence *n* = 36	Unclear *n* = 36	Depression: CSDD Multi-dimension: NPI-Q (only reported overall score)	Pre, post Rated by patients	Mann–Whitney test for change post – pre	CSDD: S -, *P* = 0.02
(48) Mainland China	>60 y/o ADRD patients from hospitals *n* = 58	Randomized study	8-week 8-session individual life story book *n* = 29	Usual care *n* = 29	Depression: CSDD Quality-of-life: DQOL	Pre, post, 12-week f/u Rated by patients	*t*-test	post: CSDD: S -, *t* = 3.84, *P* = 0.00 DQOL: S +, *t* = 5.72, *P* = 0.00 f/u: CSDD: S -, *t* = 4.33, *P* = 0.00 DQOL: S +, *t* = 4.68, *P* = 0.00
(54) Taiwan	≥65 y/o ADRD patients from nursing homes *n* = 102	RCT	8-week 8-session group reminiscence *n* = 51	Unclear *n* = 51	Depression: CSDD, GDS-SF	Pre, post CSDD rated by caregivers; GDS-SF rated by patients	GLM ANCOVA	CSDD: a change favoring IG: group effect *F* = 5.13, *P* = 0.026 GDS: NS
(36) Mainland China	Older ischemic heart disease patients from a hospital *n* = 74	Randomized study	Individual narrative therapy, length unclear *n* = 37	Usual care: physical and psychological care, disease monitoring, diet, health education *n* = 37	Anxiety: SAS	Pre, post Rater unclear	*t*-test	SAS: S -, *P* < 0.05
(24) UK	ADRD patients and caregivers from mental health services for older people *n* = 488 dyads	RCT	12-week 12-session patient-caregiver reminiscence, followed by 7 monthly maintenance group sessions *n* = 268 dyads (from table)	Usual care: varied between and within centers and over time *n* = 219 dyads (from table)	Quality-of-life: QOL-AD, EQ-5D: utility score, VAS Depression: CSDD Anxiety: RAID	Pre, during intervention, post CSDD and RAID rated by caregivers; QOL-AD and EQ-5D rated by both patients and caregivers	ANCOVA	NS
(44) Taiwan	≥65 y/o ADRD patients from the geriatric division of a medical center *n* = 106	RCT	6-week 6-session group spiritual reminiscence *n* = 53	Unclear *n* = 53	Hope: Herth Hope Index Life satisfaction: Life Satisfaction Scale Spirituality: SIWB	Pre, post Rated by patients	Generalized estimating equation models	Herth Hope Index, LSS, SIWB: group x time effect *P* < 0.001
(39) USA	62–98 y/o ADRD patients from community *n* = 80	RCT	6-week 12-session individual interactive reminiscence games followed by 6-week played on patients’ own or with facility staff *n* = 32 Same as above but in group format *n* = 32	Waitlist: 12-week usual care followed by individual games *n* = 16	Mood: AMS (hostile, apathetic, sad, content, spirited) Quality-of-life: QOL ladder	Pre, post (6-week), 12-week f/u AMS rated by caregivers, QOL rated by patients	Raw data provided by authors as requested	Not applicable

*A “quasi-experimental” study refers to one that the randomization could not be ruled out, but authors used “quasi-experimental” in text. **A “randomized study” refers to a study in which authors did not directly describe it as an RCT, but randomization was used. AES-C, Apathy Evaluation Scale, Clinician Version; AMS, Alzheimer’s Disease and Related Dementia Mood Scale; BMMRS, Brief Multidimensional Measure of Religiousness/Spirituality; CES-D, Center for Epidemiologic Studies Depression Scale; CSDD, Cornell Scale for Depression in Dementia; DQOL, Dementia Quality-of-life instrument; DRS, Minimum Data Set Depression Rating Scale; GAI, Geriatric Anxiety Inventory; GDS, Geriatric Depression Scale; GDS-12R, GDS (Residential); GDS-SF, GDS Short Form; HAMD, Hamilton Depression Rating Scale; LSI-A, Life Satisfaction Index-A; MOSES, Multidimensional Observation Scale for Elderly Subjects; MSAS-SF, Memorial Symptom Assessment Scale – Short Form; NOSIE, Nurses’ Observation Scale for Inpatient Evaluation; NPI-Q, Neuropsychiatric Inventory Questionnaire; PANAS, Positive and Negative Affect Schedule; QOL-AD, Quality-of-life in Alzheimer’s Disease; QOL ladder, Cantril QoL ladder; RAID, Rating Anxiety In Dementia; RSE, Rosenberg Self-Esteem Scale; SAS, Zung Self-Rating Anxiety Scale; SDS, Zung Self-Rating Depression Scale; SIWB, Spirituality Index of Well-Being; SWLS, Satisfaction with Life Scale; WIB, Well-being/Ill-being Scale.

In terms of the intervention details, apart from five studies that did not provide the information on the intervention length (35–37, 41, 42), the length ranged from 4 weeks to 10 months, with more than half (*n* = 23) fell between six to 12 weeks. The number of sessions ranged from 4 to 24. Sixteen interventions were conducted individually with an older adult; 12 were in groups of older adults; three were attended by older adults together with their caregivers (24, 34, 37); one study did not specify (43). For the remaining two studies, in Thorgrimsen et al. (38), caregivers first participated in the intervention for 11 weeks, followed by 7-week older adult-caregiver dyads participating together; in 39, there were two intervention groups, with the content being the same but in the individual and the group format, respectively.

Researchers claimed the reminiscence was used in 27 studies, including one that used the reminiscence gaming on a device (39), and one that used the “spiritual reminiscence” (44). For others, three used life review (42, 45, 46), three used narrative therapy (35, 36, 47), and one used life-story book (48). Control groups received usual care in 20 studies (including one waitlist, (39)), passive control activities in three studies such as weekly calls and conversations on topics not related to life review (37, 49, 50), no intervention in three studies (38, 42, 51), and other activities relevant to the interventions in two studies (46, 52). Three studies did not provide control group information (44, 53, 54). In the remaining three studies, two control groups were used. In Lai and Kayser-Jones (55), one group received passive control and the other did not receive any treatment; in Mei et al. (34), one group was the reminiscence received only by caregivers, and the other was usual care group; in Serrani Azcurra (43), one was the active control (i.e., counseling) and the other was the passive control.

### Psychospiritual outcomes and measurement time points

Eleven psychospiritual outcomes were measured. Depression was measured in more than half of studies (*n* = 24), QOL measured in 11, and anxiety in five. Life satisfaction was measured in three studies. Mood, apathy, spirituality, and general well-being were measured in two studies. Self-esteem, meaning in life, and hope were measured in one study. Please see Table 1 for detailed information.

Additionally, four multi-dimensional measurement tools were used in several studies. They were not included in the meta-analysis, either because only a total score was calculated, or because the same concept was measured in the study using a more widely used scale. These tools included Neuropsychiatric Inventory Questionnaire (NPI-P) (measured in four studies), which measures a variety of symptoms such as delusions and hallucinations but also psychospiritual outcomes such as depression and anxiety; Multidimensional Observation Scale for Elderly Subjects (MOSES) (two studies), which includes five domains: self-care, disorientation, depression, irritability, and withdrawal; Nurses’ Observation Scale for Inpatient Evaluation (NOSIE) (one study); and Memorial Symptom Assessment Scale – Short Form (MSAS-SF) (one study), which includes four parts: physical symptoms, physical bother, emotional symptoms, and emotional bother.

A majority of studies (*n* = 24) had pre- and post-intervention assessments; others had during-intervention, or follow-up assessments, or both. However, the follow-up assessments were conducted at a variety of different time points (e.g., 1 month, 6 weeks, and 3 months after the intervention), which made it difficult to conduct meta-analyses for results of follow-up assessments.

Regarding participants’ diseases/conditions, participants in most studies (*n* = 27) were ADRD patients, four were stroke patients (20, 32–34), two were patients with ischemic heart disease (35, 36), and one were general palliative care patients (37). The number of participants ranged from 11 to 488, with most studies (*n* = 29) had fewer than 100 participants. Participants in most studies (*n* = 28) were recruited from certain types of institutions, including hospitals, nursing homes/old age homes/care homes or long-term care facilities in general, assisted living facilities, and medical centers. Participants in three studies were community-dwelling older adults (20, 38, 39). Participants in two studies were recruited from multiple sources (37, 40), and one study unclear (34).

Most studies (*n* = 29) reported significant between-group results in at least one outcome domain at one time point. For outcomes measured only in one study, Wu and Koo (44) found that the interaction term between group and time for hope was significant (*P* < 0.001), indicating the changes over time in hope was different between the intervention and the control group. No significant differences were found for self-esteem in Morgan (42) or for meaning in life in Allen et al. (37). Meta-analysis results for other outcomes are reported in one section below.

### Risk of bias

**FIGURE 2:**
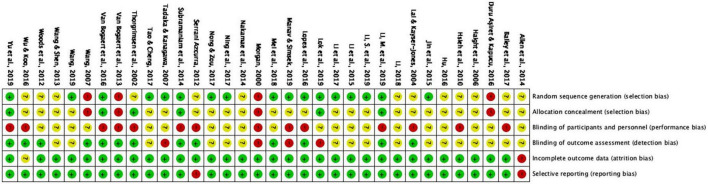
Risk of bias summary.

Risks of bias were summarized in Figure 2. Regarding random sequence generation, 17 studies were judged to be at low risk, which used drawing lots, random numbers (using a table or envelopes), and randomization software. Four studies were judged to be at high risk in this domain. Specifically, in Duru Aşiret and Kapucu (49); Van Bogaert et al. (40), and Wang (54), odd-number participants were assigned into the control group and even numbers into the intervention group. In Morgan (42), the initial participants were randomly assigned alternatively to the groups, while subsequent participants were allocated to groups using randomization by minimization.

For allocation concealment, six studies were judged to be at low risk, including five that used sealed envelopes (38, 39, 51, 53, 56), and one study in which the whole randomization process was conducted by an accredited clinical trials unit (46). A majority of studies (*n* = 24) did not report information on allocation concealment, and the remaining four studies (40, 42, 49, 54) were judged to be at high risk.

Regarding blinding participants and interventionists, 14 studies were rated as high risk and 20 studies as unclear, with no studies judged to be at low risk. Meanwhile, 13 studies were judged as low risk for blinding outcome assessment, by using personnel not included in the intervention process. In four studies (42, 50, 51, 57), evaluators were not blind to group assignment. Seventeen studies did not provide information in this domain.

One study (37) was judged to be at high risk for incomplete outcome data because of a high dropout rate at the baseline assessment (41% dropped out), and the dropout rate was higher in intervention group than in control group. One study (44) did not provide information for dropouts and thus rated as unclear. The remaining studies were all rated as low risk in this domain. Lastly, two studies were judged as high risk for selective reporting. Allen et al. (37) did not report results for follow-up assessments, and Serrani Azcurra (43) did not report anxiety and depression scores that were stated to have been measured, based on the article’s methods section.

### Meta-analysis results

We performed meta-analysis with outcomes that were measured in at least two studies. We found that most of our meta-analyses showed considerable heterogeneity, and further performed sensitivity and subgroup analyses. However, heterogeneity was not improved. We reported sensitivity and subgroup analyses for depression as an example. Subgroup analyses for other outcomes are available upon request.

### Depression

**FIGURE 3:**
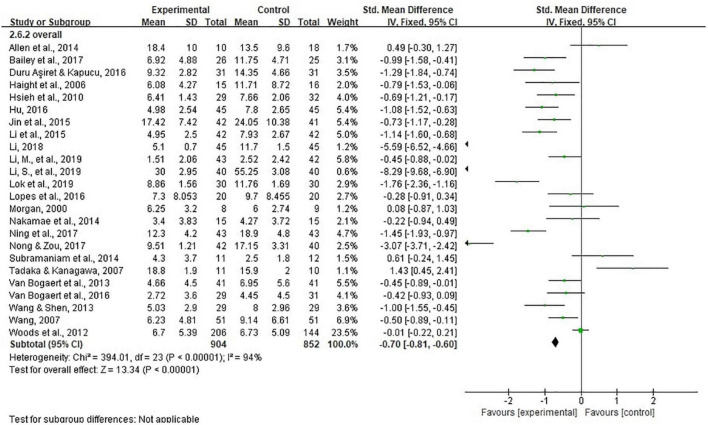
Effect size of the intervention group versus the control group on depression rating scores.

Twenty-four studies representing 1,756 participants reported data on depression and were pooled for a meta-analysis using the random-effects model. Considerable heterogeneity was identified (I^2^ = 94%, *P* < 0.00001), while results showed that there were significant standardized mean differences in favor of life review intervention compared with controls for depression [SMD = −1.07, 95% CI (−1.52, −0.63), *P* < 0.00001] (Figure 3). Sensitivity analysis was performed but high heterogeneity still existed. Based on the forest plot, there were two outliers. After removing Li et al. (47), I^2^ was 92% [SMD = −0.84, 95% CI (−1.22, −0.45)]; after removing Li (33), I^2^ was 92% [SMD = −0.88, 95% CI (−1.27, −0.49)].

We also performed subgroup analyses with various possible reasons for heterogeneity (e.g., study quality, cognition, measurement tool, outcome rater, control group, country/region, intervention weeks, number of intervention sessions) (Table 2). However, heterogeneity remained high in subgroups except for two studies in UK [I^2^ = 53%, SMD = −0.05, 95% CI (−0.51, 0.41), *P* = 0.83], and two studies in Taiwan [I^2^ = 0%, SMD = −0.57, 95% CI (−0.88, −0.26), *P* = 0.0004]. Meanwhile, a significant difference (*P* = 0.0005) was found in terms of country/region where the study was conducted. Although with considerable heterogeneity (I^2^ = 97%), pooled results of eight studies in China showed a SMD of −2.39 [95% CI (−3.37, −1.42), *P* < 0.00001], an absolute value much larger than those in other countries and regions.

**TABLE 2:** Subgroup analyses for depression.

	Description	# of studies	I^2^	SMD [95% CI]	*P* (overall effects)	*P* (group difference)
Low random allocation risk		11	95%	−1.32 [−2.13, −0.51]	0.001	
Excluding four or more “high” + “unclear” in ROB appraisal		14	95%	−1.07 [−1.65, −0.48]	0.0003	
ADRD patients		15	89%	−0.78 [−1.16, 0.41]	0.0001	
Same instrument	CSDD	13	80%	−0.68 [−0.97, −0.40]	0.00001	0.13
	GDS	4	93%	−1.35 [−2.43, −0.27]	0.01	
	HAMD	3	98%	−2.54 [−4.69, −0.40]	0.02	
Outcome rater	Patients	11	88%	−0.78 [−1.30, −0.25]	0.004	0.05
	Caregivers	4	82%	−0.05 [−0.54, 0.43]	0.83	
Control group	Usual care	16	96%	−1.43 [−2.04, −0.82]	0.00001	0.61
	No intervention	2	90%	−0.88 [−2.68, 0.92]	0.34	
Country/Region	China	8	97%	−2.39 [−3.37, −1.42]	0.00001	0.0005
	UK	4	53%	−0.05 [−0.51, 0.41]	0.83	
	USA	2	89%	−0.28 [−1.72, 1.17]	0.71	
	Taiwan	2	0%	−0.57 [−0.88, −0.26]	0.0004	
	Japan	2	86%	0.57 [−1.05, 2.19]	0.49	
Intervention weeks	>10	8	93%	−0.85 [−1.46, −0.23]	0.007	0.22
	≤10	13	95%	−1.47 [−2.24, −0.69]	0.0002	
Number of sessions	>10	11	91%	−0.75 [−1.23, −0.27]	0.002	0.10
	≤10	11	96%	−1.63 [−2.56, −0.70]	0.0006	

### Anxiety

**FIGURE 4:**
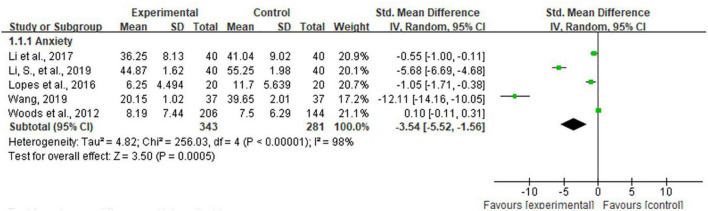
Effect size of the intervention group versus the control group on anxiety rating scores.

Five studies representing 624 participants reported data on anxiety and were pooled for a meta-analysis using the random-effects model. Considerable heterogeneity was identified (I^2^ = 98%, *P* < 0.00001), while results showed that there were significant standardized mean differences in favor of life review intervention compared with controls for anxiety [SMD = −3.54, 95% CI (−5.52, −1.56), *P* = 0.0005] (Figure 4).

### QOL

**FIGURE 5:**
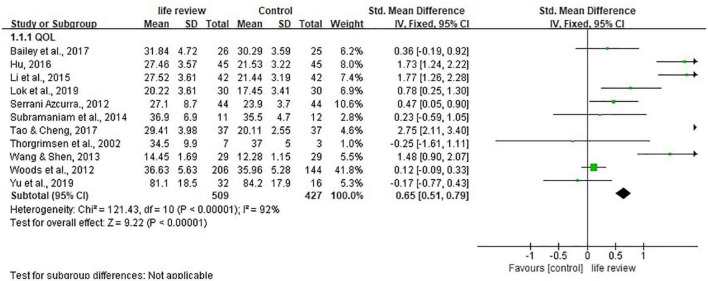
Effect size of the intervention group versus the control group on quality-of-life rating scores.

Eleven studies representing 936 participants reported data on QOL. Random-effects model showed considerable heterogeneity (I^2^ = 92%, *P* < 0.00001) and significant standardized mean differences was in favor of life review intervention [SMD = 0.87, 95% CI (0.34, 1.41), *P* = 0.001] (Figure 5).

### Life satisfaction

**FIGURE 6:**
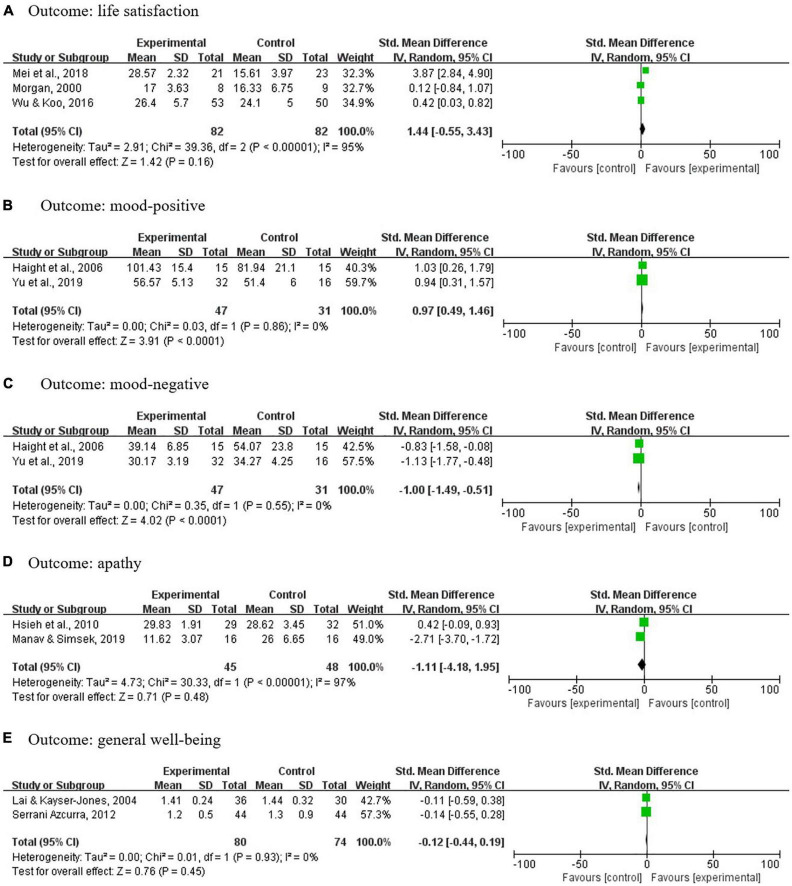
Meta-analyses results on life satisfaction, mood, apathy, and general well-being.

Three studies (34, 42, 44) with a total of 164 participants were pooled using the random-effects model. Results showed considerable heterogeneity (I^2^ = 95%, *P* < 0.00001) and no significant differences between the intervention and the control groups in life satisfaction (*P* = 0.16) (Figure 6A).

### Mood

Mood was measured in two studies (39, 45) using the Alzheimer’s Disease and Related Dementia Mood Scale, with five domains (i.e., hostile, apathetic, sad, contented, and spirited) that are categorized into positive (including contended and spirited) and negative moods (including hostile, apathetic, and sad). Meta-analyses with 78 participants showed low heterogeneity and significant standardized mean differences in favor of life review intervention in increasing one’s positive moods and decreasing negative moods [positive: I^2^ = 0%, SMD = 0.97, 95% CI (0.49, 1.46), *P* < 0.0001; negative: I^2^ = 0%, SMD = −1.00, 95% CI (−1.49, −0.51), *P* < 0.0001] (Figures 6B, C).

### Apathy

Two studies (50, 58) with a total of 93 measured the apathy outcome, an indicator of motivation to get things done. Due to different score directions, scores in Manav and Simsek (50) were reversed to make them comparable to the other study. Results showed considerable heterogeneity (I^2^ = 97%, *P* < 0.00001) and no significant differences between the intervention and the control groups in apathy (*P* = 0.48) (Figure 6D).

### Spirituality

Two studies (37, 44) measured spirituality well-being; however, results could not be pooled for the meta-analysis. In Allen et al. (37), four subscales (daily spiritual experience, forgiveness, meaning, abandonment by God) with different score directions of Brief Multidimensional Measure of Religiousness and Spirituality (BMMRS) were used. As the instrument’s development teams did not suggest combining the subscale scores (59), we did not generate a combined score to perform the meta-analysis. Regarding the results, Allen et al. (37) reported that, compared to the control group, intervention group expressed more forgiveness [*F*(1,25) = 5.24, *P* = 0.031; partial eta-squared = 0.17] and less abandonment by God [*F*(1,25) = 4.88, *P* = 0.037; partial eta-squared = 0.16]. While for Wu and Koo (44), spirituality well-being score increased in the intervention group but decreased in the control group, with a significant time x group interaction term (*P* < 0.001).

### General well-being

Two studies (43, 55) with 154 participants measured participants’ general well-being and results were pooled for meta-analysis. With a low heterogeneity (I^2^ = 0), no significant differences were found between the intervention and control groups in general well-being (*P* = 0.45) (Figure 6E).

### Publication bias

**FIGURE 7:**
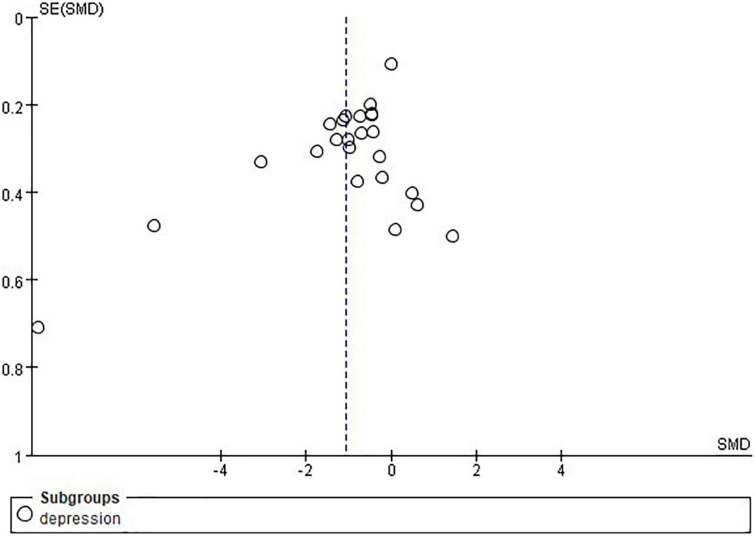
Funnel plot of the included studies on depression.

**FIGURE 8:**
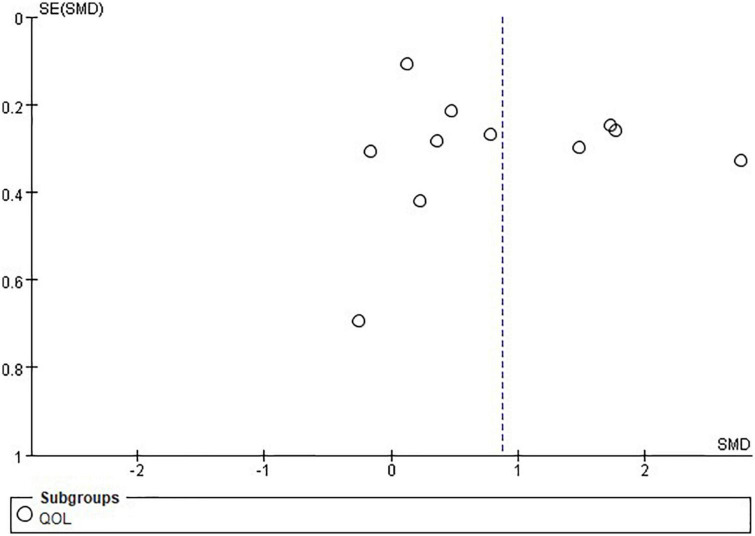
Funnel plot of the included studies on quality-of-life (QOL).

Publication bias was assessed for depression and QOL through visually inspecting asymmetry in the funnel plots. The funnel plot showed asymmetry indicating potential publication bias was present in this review, which might have influenced the results (Figures 7, 8).

## Discussion

This systematic review and meta-analysis study examined the effects of life review on psychospiritual outcomes among older adults with LTI. A total of 34 studies representing 2,752 participants were identified. Our results indicated that life review could benefit older adults with LTI in decreasing anxiety, depression, and negative mood, and enhancing their positive mood and QOL. Meanwhile, many other psychospiritual outcomes were measured less frequently, and high-quality studies were lacking.

Previous meta-analyses for older adults also showed the effects of life review on decreasing depression (15, 16). However, the effects of life review on QOL were not significant in Lan et al. (16). It could be because of the small number of studies included for meta-analyses in Lan et al. (16): only two studies were pooled for the effects of QOL. It could also be because, while their study focused on the general older adult population, our study focused on older adults with LTI, which is a group of individuals who have been undergoing great distress because of their physical and psychological conditions (3), and thus the therapeutic benefits of life review could be more likely to manifest among this population. Life review interventions encourage older people to remember and talk about their life histories. In doing so, older adults with LTI recognize their strengths and resilience and feel satisfaction from knowing that their lives have meaning. In other words, life review brings the resilience, hardiness, and wisdom of life experiences and lessons learned; the more, the less vulnerability and risk of illnesses. It is noteworthy that during the life review sessions, both positive and negative life experiences may be brought about. While positive experiences make participants feel delighted and fulfilled, adverse life events also have meanings. By reviewing negative ones, older adults with LTI may re-evaluate the experience and come to peace with the past and the present, thus increasing their resilience to cope with difficult, stressful, and traumatic situations.

In addition to depression, anxiety, and QOL, measured in at least five studies, other psychospiritual outcomes included life satisfaction, mood, apathy, spirituality, self-esteem, meaning in life, hope, and general well-being. Only a small number of studies measured them; thus, it is less possible to draw a firm conclusion about the effectiveness of life review. However, these concepts were hugely important for older adults living with LTI: even with a heightened awareness of mortality, people living with LTI would try to have a positive outlook on life and tend to focus more on family and relationships (7). They would also respond to loss and suffering by becoming certain and taking control of the uncontrollable (4). Additionally, many people with LTI reported relying on religion to cope with their illnesses (2). Thus, we call for more research on interventions for older adults living with LTI to include more psychospiritual outcome measures, such as hope and self-esteem, to understand better this population’s remarkable story of growth and transformation through adversity. Life review, by its name, could be a valuable tool to discuss their life experiences and solicit their thoughts and feelings for the past, present, and future.

In this review, high-quality evidence is lacking, and only a few studies were judged to be at low risk in at least five risk of bias domains. Although half studies were rated as low risk in the random sequence generation, only a few reported adequate allocation concealment. No study was rated as low risk in blinding of participants and personnel; however, this is indeed difficult to achieve in psychosocial interventions, as interventionists and participants are naturally aware of the intervention activities. However, more than half of the studies did not report blinding of outcome assessment, or outcome assessors were not blinded. This could generate bias as unblinded assessors might exaggerate the intervention effects. In the future, studies with a more rigorous study design should be implemented, paying special attention to random sequence generation, allocation concealment, and blinding of outcome assessment.

This study has several limitations. First, we only included English and Chinese literature due to the team’s language capacity, and thus excluded potentially useful information written in other languages. Second, we used the WHO’s list of leading causes of death as criteria for LTI. However, people could undergo different stages of an illness, and patients with certain illnesses or in specific stages (such as early stages of cancer) do not face impending death. Third, studies that used four multi-dimensional measurement tools were not included in the meta-analysis. This could bring a risk of bias due to missing results in synthesis. Fourth, this study might be limited by the selected databases. Although the investigators included the most widely used English and Chinese databases, it remains possible that some works, particularly unpublished studies conducted in other countries, were not located and examined. Finally, our meta-analysis results also showed considerable heterogeneity, which could be because of great variations in terms of intervention format, length, setting, study quality, and other study characteristics; however, we believe this study serves as one of the first steps for the team to go deeper into researching the intervention effects on psychospiritual outcomes, and thus in the current stages, studies including this one are still exploratory.

Future research implications are as follows. First, more well-designed RCT studies are warranted to evaluate the psychospiritual effects of life review interventions among older adults with LTI. The outcome should include not only depression, anxiety, and QOL, but also other psychospiritual outcomes crucial to one’s holistic well-being. We also call for more rigorous design, especially high-quality designs that minimize biases in domains of random sequence generation, allocation concealment, and blinding of outcome assessment. In addition, studies with larger sample sizes are necessary to clarify the efficacy. Finally, future research can include qualitative studies to obtain the opinions of participants with different backgrounds on the optimal time, length, number of sessions, and other characteristics of life review interventions.

## Conclusion

Despite some limitations, the study findings support a positive effect of life review on psychospiritual outcomes among older adults with LTI. Healthcare providers in different settings should continue to utilize life review for this population. They should measure and monitor their effects on different psychospiritual outcomes, to investigate how these interventions help them cope with challenges, increase resilience, and maintain hope and a deeply experienced sense of self-identity in the intersection of old age and life-threatening illness.

## Data availability statement

The raw data supporting the conclusions of this article will be made available by the authors, without undue reservation.

## Author contributions

ML, YW, and IC designed the study. ML and YW conducted the title and abstract screening. ML drafted the manuscript. All authors extracted the data and attended the group meetings to reach a consensus, critically reviewed it and provided the feedback, and contributed to the article and approved the submitted version.
